# Supplementary material for: Global state and potential scope of investments in watershed services for large cities
Source: Nat Commun. 2018 Oct 22;9:4375. doi: 10.1038/s41467-018-06538-x (PMC6197214; doi:10.1038/s41467-018-06538-x)
Supplement: Supplementary file 1 — Supplementary Information [file 41467_2018_6538_MOESM1_ESM.pdf]

Supplementary Information Cover Page

Title of the Manuscript: Global State and Potential Scope of Investments in Watershed Services for Large Cities

First Author's surname: Romulo et al.

Supplementary Figures

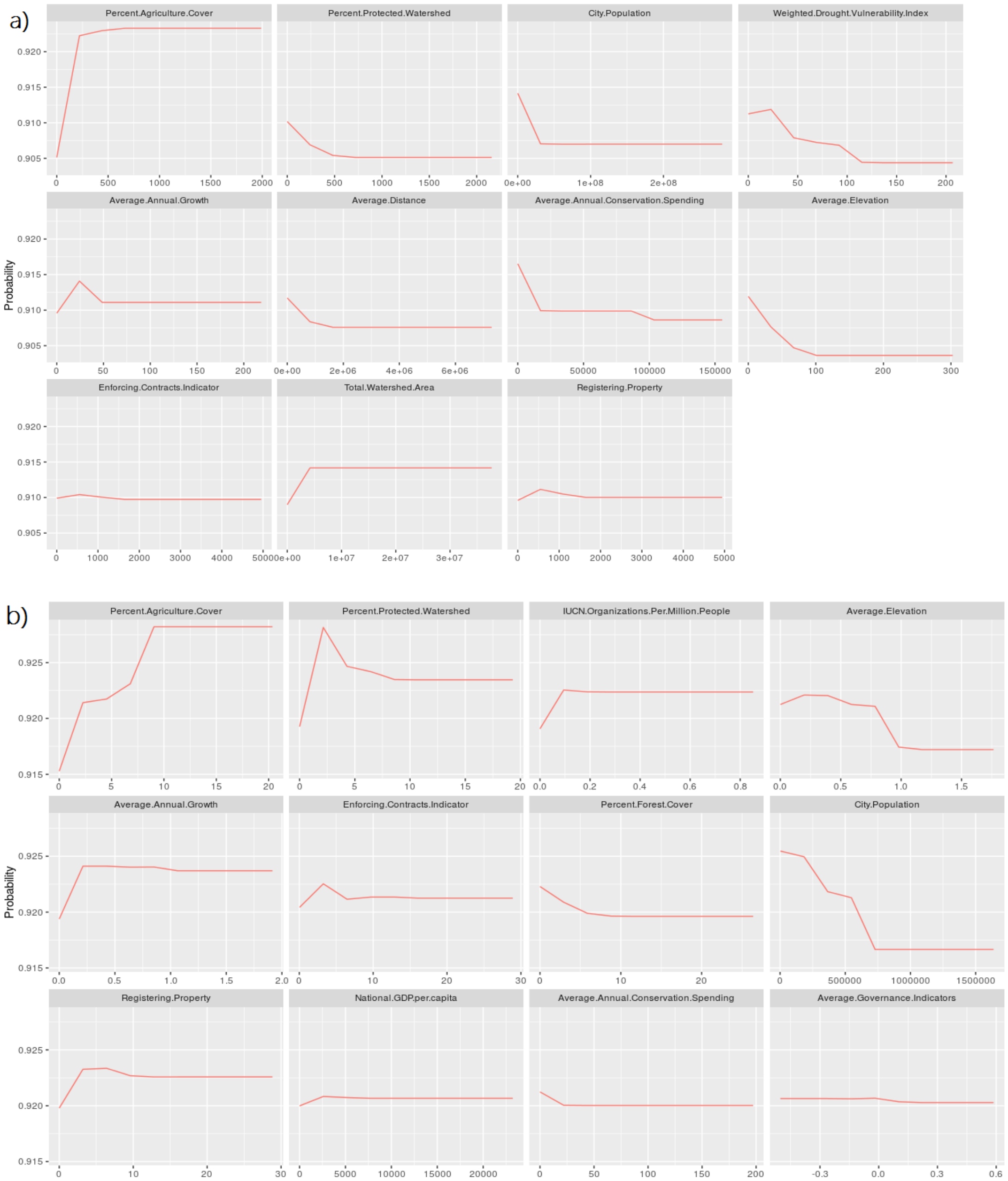

Supplementary Figure 1: Partial dependence plots of important enabling conditions variables for (a) Global Cities, and (b) Non-USA Cities. Line shapes below explain directionality of prediction ability and thresholds, but scale and numbers do not represent accuracy or prediction. The variables included are two subsets, (a) and (b), because we provide partial dependence plots only for those conditions ranked important in each random forest model. Plots are displayed in the same rank order as conditions in Figure 3 in main manuscript, from most to least important (left to right). Each plot displays the relative contribution of the variable within the model for predicting the presence of an IWS program. The X axis displays the weighted model values for each variable. The Y axis provides the probability, or predicted value, of IWS presence for given values of X, all other variables held constant within the model.

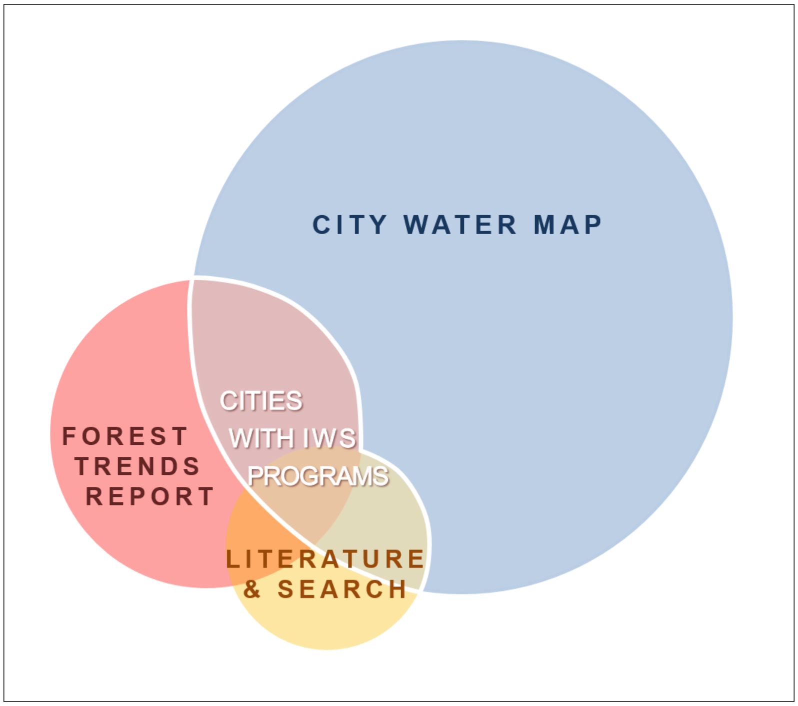

Supplementary Figure 2: Venn diagram of identification of cities with IWS programs.

The resulting database of cities, watersheds, and IWS program information contains 416 cities globally, 48 of which have a IWS program pertaining to drinking water. Of the 48 large cities with programs, 18 are in the United States and 30 are in other countries. These 48 programs represent 239,688,129 people, or about 3% of the total world population, or about 20% of the population living in large cities (about 1.2 billion people in our database).

Supplementary Tables

Supplementary Dataset 1 (Separate File): Representative data descriptions and relationships to Enabling Conditions and Bins from Huber Stearns et al. 2017.

Supplementary Dataset 2 (Separate File): Enabling conditions values and rankings for top 5 conditions from the Non-USA cities model. Cities outside of the USA divided into top or bottom medians (threshold values indicated in italics) and then were selected (1) based on the enabling condition relationship described by the partial dependence plots (Supplementary Figure 1).

Supplementary Dataset 3 (Separate File): Representative data for all cities.

Supplementary Table 1: Scale and Selection of Enabling Condition Variables. The attributes used in this analysis are specific to either cities and their water sources or the country in which the city is found. Watershed attributes for both surface and groundwater sources are linked to each city on the basis of City Water Map diversion ID and diversion type. Binary variables (IWS Program and Located in USA) were used to group cities prior to analysis. Variables in bold were used in random forest model development. Variables in grey **were not used in the analysis based on observed correlations with other variables. A final data table with all representative data is provided as Supplementary Table 3.**

City Level Data	Country Level Data
Analysis groups	**Average Annual Growth**
**IWS Program (Yes/No)**	**Average Governance Indicators**
**Located in USA (Yes/No)**	**Conservation Spending**
Water source attributes	Doing Business Indicators
**Average Distance**	**Enforcing Contracts Indicators**
**Average Elevation**	GCR Property Rights
**City Population**	National GDP
Distance	**National GDP per Capita**
Elevation Range	**Number of IUCN Organizations Per Million People**
International Boundary Crossings	**Registering Property**
Mean Groundwater Drought Vulnerability	
Mean Surface Water Drought Vulnerability	
Number of Diversions	
**Percent Agricultural Cover**	
**Percent Forest Cover**	
**Percent Protected Area**	
Percent Surface Water Withdrawals	
**Quantity of Withdrawals**	
**Total Watershed Population***	
**Water Quantity**	
**Watershed Area**	
**Watershed Population Density**	
**Weighted Drought Vulnerability**	
*In surface watersheds only. All other variables include above and belowground sources.	

.

Supplementary Table 2: Land cover classifications and groups used for calculating water source land cover.

Code	Class	Type
0	Water	Water
1	Evergreen Needleleaf forest	Forest
2	Evergreen Broadleaf forest	Forest
3	Deciduous Needleleaf forest	Forest
4	Deciduous Broadleaf forest	Forest
5	Mixed forest	Forest
6	Closed shrublands	Natural non-forest
7	Open shrublands	Natural non-forest
8	Woody savannas	Natural non-forest
9	Savannas	Natural non-forest
10	Grasslands	Natural non-forest
11	Permanent wetlands	Natural non-forest
12	Croplands	Agricultural and pastoral
13	Urban and built-up	Urban
14	Cropland/Natural vegetation mosaic	Agricultural and pastoral
15	Snow and ice	Natural non-forest
16	Barren or sparsely vegetated	Natural non-forest
254	Unclassified	None
255	Fill Value	None

Supplementary Table 3: WUP class sizes (UNPD 2014):

CLASS	Population Range
A	300 000 to 499,999
B	500 000 to 999,999
C	1 million to 4,999,999
D	5 million to 9,999,999
E	10 million or more

Supplementary Table 4: Selection and justification for removing or retaining variables with a correlation coefficient > 0.7 calculated using Spearman correlation coefficient.

Pairwise Correlation	Correlation Coefficient	Selection and Justification
(1) Average Distance<br><br>(2) Distance	corr(1,2) = 0.864671	The average distance measure was kept because the distance measure represented the minimum distance between the water source and beneficiaries, while the average distance provides a better proxy for the distance between water users and source water.<br>Distance: Euclidean distance (m) to closest diverter calculated from CWM<br>KEEP Avg_Distance:Average distance (m) of all city diverters calculated from CWM<br>REMOVE Distance
(1) Weighted Drought Vulnerability Variable<br>(2) Mean Surface Water Drought Vulnerability<br>(3) Mean Groundwater Drought Vulnerability	corr(1,2) = 0.995444<br>corr(2,3) = 0.796997<br>corr(1,3) = 0.897232	The weighted drought vulnerability measure was kept because it is calculated using the values from the average surface water and groundwater drought vulnerability.<br>DRT_WGT: Weighted drought variable that takes into account both surface water and groundwater drought vulnerability index.<br>SWDRT_Mean: Mean drought vulnerability of source surface water. Based on drought vulnerability index ranging from 1 to 10 where 10 is highest risk<br>GWDRT_Mean: Mean drought vulnerability of source groundwater. Based on drought vulnerability index ranging from 1 to 10 where 10 is highest risk<br>REMOVE SWDRT_Mean and GWDRT_Mean<br>KEEP DRT_WGT because it is calculated by the other two.
(1) Average Elevation<br>(2) Elevation Range	corr(1,2) = 0.722886	Average Elevation was used because range gives no indication of absolute elevation.
(1) Conservation Spending<br>(2) Current GDP	corr(1,2) = 0.935175	Current GDP was removed because other GDP metrics were available and included in some of the World Bank indices.<br>Cons_spending: Estimated country-level spending on conservation. Data for total average annual spending (in $ US million 2005) from 2001-2008, including all flows of funding: international donors, domestic governments, trust funds, and self-funding via user payments<br>Natl_GDP_Curr: Gross Domestic Product using official conversion to US$ as of 09 January 2016
(1) GDP per capita<br>(2)GCR Property Rights	corr(1,2) = 0.79643	National per capita GDP was kept as a measure of economic growth. The GCR property rights index was removed because other property rights proxy variables exist.
(1) World Bank Aggregate Governance Indicator<br>(2) Doing Business Average Indicator<br>(3) Enforcing Contracts Indicator<br>(4) Average Annual Growth<br>(5) GDP per capita<br>(6) Conservation spending	corr(1,2) = 0.820512<br>corr(1,3) = 0.753173<br>corr(1,5) = 0.80704<br>corr(1,6) = 0.715973<br>corr(2,3) = 0.753173<br>corr(2,5) = 0.878658<br>corr(3,5) = 0.729046<br>corr(4,5) =-0.76473	The calculated Doing Business Average index score and almost all subscores (save those listed below) were removed because of high correlation with the World Bank Aggregate Governance Indicators.<br><br>Enforcing Contracts was included as a proxy for institutional capacity to administer IWS agreements with landowners. The indicator is based on time/cost metrics for resolving commercial disputes and the structure and quality of court systems.<br><br>Registering Property was kept as a proxy of the transaction costs associated with making agreements about the use of land. The indicator is a measure of the time, cost, and bureaucratic logistics of transferring property title and using real estate as collateral for lending.

Supplementary Table 5: Variance Inflation Factors for all cities and non-USA city subset of final 17 variables. Bolded variables exceed the VIF selection threshold of 3.

Enabling Condition Representative Data	All cities	Non- USA cities
Average Elevation	1.5733	**3.2665**
Average Annual Conservation Spending	**4.3206**	**7.0211**
Average Annual Growth 1994-2014	2.4129	**4.3363**
Average Distance	1.1561	1.8876
Average Governance Indicators	**5.9668**	**6.9895**
Average Watershed Population Density	1.1420	1.2256
City Population	1.3692	**4.1986**
Enforcing Contracts Indicator	2.3569	**13.5216**
Number of IUCN Organizations per Million People	1.8299	3.0302
National GDP per capita	9.1247	12.6280
Percent Agricultural/Pastoral Cover	1.9525	1.9429
Percent Forest Cover	1.6673	2.8831
Percent Protected Watershed	1.3920	2.1738
Registering Property Indicator	2.2770	17.4181
Total Diversion Volume	1.1378	1.0179
Total Watershed Area	1.2961	1.1341
Weighted Drought Vulnerability Index	1.4708	3.0189

Supplementary Table 6: Correlation Coefficients of final 17 indicator variables (representative data) used in the random forest model. Coefficient values >0.7 are in bolded text.

	Weighted Drought Vulnerability Index	Average Elevation	Water Area	Average Watershed Population Density	Total Diversion Volume	Average Distance	Percent Forest Cover	Percent Agriculture Cover	Percent Protected Watershed	Average Annual Conservation Spending	IUCN Organizations Per Million People	City Population	Average Governance Indicators	Enforcing Contracts Indicator	National GDP per Capita	Average Annual Growth
Weighted Drought Vulnerability Index	**1.000**	0.145	0.027	0.144	-0.040	0.114	-0.408	-0.034	-0.011	0.019	-0.149	-0.120	-0.069	-0.235	-0.154	0.124
Average Elevation	0.145	**1.000**	-0.115	0.112	0.196	0.144	0.292	-0.417	0.238	-0.106	0.008	0.026	-0.031	0.031	0.019	0.019
Water Area	0.027	-0.115	**1.000**	0.129	0.013	0.178	-0.038	0.000	0.007	0.086	-0.029	0.174	-0.018	0.098	0.042	0.008
Average Watershed Population Density	0.144	0.112	0.129	**1.000**	0.144	0.214	0.041	-0.049	0.040	-0.031	0.063	0.024	0.055	0.171	0.077	-0.051
Total Diversion Volume	-0.040	0.196	0.013	0.144	**1.000**	0.330	0.038	-0.045	0.125	-0.268	0.049	0.307	-0.091	0.064	-0.025	-0.033
Average Distance	0.114	0.144	0.178	0.214	0.330	**1.000**	0.020	-0.092	0.080	-0.162	0.065	0.409	-0.040	0.142	0.009	-0.020
Percent Forest Cover	-0.408	0.292	-0.038	0.041	0.038	0.020	**1.000**	-0.323	0.151	0.269	0.136	0.019	0.283	0.402	0.390	-0.297
Percent Agricultural Cover	-0.034	-0.417	0.000	-0.049	-0.045	-0.092	-0.323	**1.000**	-0.205	-0.090	-0.236	0.030	-0.116	-0.181	-0.242	0.224
Percent Protected Watershed	-0.011	0.238	0.007	0.040	0.125	0.080	0.151	-0.205	**1.000**	-0.063	0.229	0.027	0.175	0.058	0.124	-0.337
Average Annual Conservation Spending	0.019	-0.106	0.086	-0.031	-0.268	-0.162	0.269	-0.090	-0.063	**1.000**	0.119	-0.308	**0.716**	0.447	**0.707**	-0.453
IUCN Organizations Per Million People	-0.149	0.008	-0.029	0.063	0.049	0.065	0.136	-0.236	0.229	0.119	**1.000**	-0.183	0.571	0.425	0.567	-0.666
City Population	-0.120	0.026	0.174	0.024	0.307	0.409	0.019	0.030	0.027	-0.308	-0.183	**1.000**	-0.281	-0.014	-0.233	0.238
Average Governance Indicators	-0.069	-0.031	-0.018	0.055	-0.091	-0.040	0.283	-0.116	0.175	**0.716**	0.571	-0.281	**1.000**	0.541	**0.807**	-0.674
Enforcing.Contracts.Indicator	-0.235	0.031	0.098	0.171	0.064	0.142	0.402	-0.181	0.058	0.447	0.425	-0.014	0.541	**1.000**	**0.729**	-0.551
National GDP per Capita	-0.154	0.019	0.042	0.077	-0.025	0.009	0.390	-0.242	0.124	**0.707**	0.567	-0.233	**0.807**	**0.729**	**1.000**	**-0.765**
Average Annual Growth	0.124	0.019	0.008	-0.051	-0.033	-0.020	-0.297	0.224	-0.337	-0.453	-0.666	0.238	-0.674	-0.551	**-0.765**	**1.000**

Supplementary Note 1: Practical implications for real-world natural resource management

Implications and implementation of research on natural resource management is critical for practitioners. We have been working with collaborators at The Nature Conservancy (a non-governmental organization) on how to use the findings from this research to improve their IWS development program. When comparing potential locations for program investment, the most important conditions can be used to evaluate where IWS programs are likely present in comparable locations. In evaluating cities for program development, those that have similar characteristics to cities that do have a program may be good candidates. Here we provide an example comparing Recife and Salvador, which are both coastal cities in Brazil. Neither currently have an IWS program according to our research, although there are other cities in Brazil that do have a program.

For this simple comparison, we will use values[1] from two enabling conditions variables[2] that were found important in the Non-USA Cities model; Percent Agriculture and Average Elevation. As the percent agriculture increases in a watershed, the likelihood that an IWS program is present increases[3]. Average Elevation of the watershed has the opposite relationship; as elevation increases, an IWS program is less likely to be present. Both Recife and Salvador are at similar elevations of 2.6 and 3.1 degrees, respectively. However, the source watershed for Recife has a much higher percentage agriculture at 34.21% compared to Salvador at 9.06%. According to our model, an IWS program is more likely present in Recife. This information is valuable when combined with local context and investment criteria to evaluate scope and expansion of IWS programs into new locations.

---

[1] All data used in this research is open source and the specific values used in our analysis can be found in Supplementary Table 3.

[2] The full list of enabling conditions (including those not tested), and the representative data used in this analysis are provided in Supplementary Table 2.

[3] Evaluation of individual variable performance within the models is provided as partial dependence plots in Supplementary Figure 1.
