# Supplementary material for: Global state and potential scope of investments in watershed services for large cities
Source: Nat Commun. 2018 Oct 22;9:4375. doi: 10.1038/s41467-018-06538-x (PMC6197214; doi:10.1038/s41467-018-06538-x)
Supplement: Supplementary file 3 — Description of Additional Supplementary Files [file 41467_2018_6538_MOESM3_ESM.pdf]

Description of Additional Supplementary Files

File Name: Supplementary Data 1
Description: Representative data descriptions and relationships to Enabling Conditions and Bins from Huber Stearns et al. 2017.

File Name: Supplementary Data 2
Description: Enabling conditions values and rankings for top 5 conditions from the Non-USA cities model. Cities outside of the USA divided into top or bottom medians (threshold values indicated in italics) and then were selected (1) based on the enabling condition relationship described by the partial dependence plots (Supplementary Figure 1).

File Name: Supplementary Data 3
Description: Representative data for all cities.

File Name: Supplementary Software
Description: Supplementary Software
