# Supplementary material for: Global state and potential scope of investments in watershed services for large cities
Source: Nat Commun. 2018 Oct 22;9:4375. doi: 10.1038/s41467-018-06538-x (PMC6197214; doi:10.1038/s41467-018-06538-x)
Supplement: Supplementary file 7 — Supplementary Software [file 41467_2018_6538_MOESM7_ESM.pdf]

IWS Code

Contents

1. Pre-Processing **1**
1.1 Functions for RF Output Plots . . . 1
1.2 Create Training and Test Data . . . 2

2.0 Random Forest Code **2**
2.1 Initial Random Forest Code with final variables after Reducing Dimensionality . . . 2
2.2 Initial Random Forest Code with final variables after Reducing Dimensionality + global city weights . . . 3
2.3 Initial Random Forest Code with final variables after Reducing Dimensionality + regional city weights . . . 5
2.4 Random Forest with SMOTE Balanced Data . . . 6
2.5 Random Forest with Downsampled Data . . . 7
2.6 Random Forest with Weighted Classes . . . 9
2.7 Partial Dependence Plots . . . 12

1. Pre-Processing

1.1 Functions for RF Output Plots

```
# Dot Chart
cforestImpDotPlot <- function(x) {
  cforest_importance <<- v <- varimpAUC(x,conditional=F)
  dotchart(v[order(v)], xlab = "Variable Importance (predictors to right of dashed vertical line are sig
  abline(v = abs(min(v)), col = "red", lty = "longdash", lwd = 2)
  abline(v = 0, col = "blue")
}
# Bar Chart
cforestImpBarPlot <- function(x) {
  cforest_importance <<- v <- varimpAUC(x,conditional=F)
  barplot(sort(v), horiz = TRUE, xlab = "Variable Importance\n(predictors to right of dashed vertical li
          las = 1)
  abline(v = abs(min(v)), col = "red", lty = "longdash", lwd = 2)
  abline(v = 0, col = "blue")
}

#make sure example trees show the node weights
update_tree <- function(x) {
  if(!x$terminal) {
    x$left <- update_tree(x$left)
    x$right <- update_tree(x$right)
  } else {
    x$weights <- x[[9]]
    x$weights_ <- x[[9]]
  }
  x
}
```

1.2 Create Training and Test Data

For factors, the random sampling occurs within each class and should preserve the overall class distribution of the data

```
install.packages("caret")
library("caret")
# For unweighted data
set.seed(3456)
CitytrainIndex <- createDataPartition(CityData$PWS_binary, p = .8,
                                  list = FALSE,
                                  times = 1)
CityTrain <- CityData[ CitytrainIndex,]
CityTest  <- CityData[-CitytrainIndex,]

# For data weighted regionally (PWS_rwgt)
set.seed(3456)
RwgttrainIndex <- createDataPartition(PWS_rwgt$PWS_binary, p = .8,
                                      list = FALSE,
                                      times = 1)
RwgtTrain <- PWS_rwgt[ RwgttrainIndex,]
RwgtTest  <- PWS_rwgt[-RwgttrainIndex,]

# For data weighted globally (PWS_gwgt)
set.seed(3456)
GwgttrainIndex <- createDataPartition(PWS_gwgt$PWS_binary, p = .8,
                                      list = FALSE,
                                      times = 1)
GwgtTrain <- PWS_gwgt[ GwgttrainIndex,]
GwgtTest  <- PWS_gwgt[-GwgttrainIndex,]
```

2.0 Random Forest Code

2.1 Initial Random Forest Code with final variables after Reducing Dimensionality

```
set.seed(66) # pick a random number

CityTrain$weights <- as.integer(CityTrain$PWS_binary+1)

# Run the Random Forest
cityfit <- cforest(as.factor(PWS_binary)~Water_Area  +
                 TOPO_MEAN +
                 WS_Dens_mean +
                 StorBL +
                 DRT_WGT    +
                 Avg_Distance +
                 Cover_PctForest +
                 Cover_PctAgpast   +
                 PctProtected  +
                 Cons_spending   +
                 Cons_IUCN         +
```

```
                 CITY_POP            +
                 Gov_Index +
                 Enforcing.Contracts.Indicator  +
                 Natl_GDP_per_capita_Curr   +
                 AvgAnnGrowth     +
                 Registering.Property,
               data=CityTrain,
               weights=CityTrain$weights,
               controls=cforest_unbiased(ntree=4000,mtry=4,trace=T))
cforestImpDotPlot(cityfit)
```

Model Evaluation

```
### Model Evaluation
#Steps for ROC Curve and AUC calculation
# Use the model to predict the evaluation.
CityTest$prediction <- predict(cityfit, newdata=CityTest)
# Extract the class probabilities.
CityTest$probabilities <- 1- unlist(treeresponse
                                     (cityfit,
                                     newdata=CityTest),
                                     use.names=F)[seq(1,nrow(CityTest)*2,2)]
# Plot the performance of the model applied to the evaluation set as
# an ROC curve.
require(ROCR)
cityfit.pred <- prediction(CityTest$probabilities, CityTest$PWS_binary)
cityfit.perf <- performance(cityfit.pred,"tpr","fpr")
cityfit.auc.perf = performance(cityfit.pred, measure = "auc")
cityfit.auc.perf@y.values # this just prints out the AUC so I can look at it real quick
plot(cityfit.perf,
     main=paste('ROC CURVE, AUC:',round(cityfit.auc.perf@y.values[[1]],digits = 4)),
     print.thres=TRUE,
     colorize=T)
abline(h=1,col='blue',lwd=2)
abline(h=0,col='red',lwd=2)
```

Initial Random Forest Code with final variables after Reducing Dimensionality AUC: 0.7007

2.2 Initial Random Forest Code with final variables after Reducing Dimensionality + global city weights

```
Set.seed(66)

GwgtTrain$weights <- as.integer(GwgtTrain$PWS_binary+1)

gwgtfit <- cforest(as.factor(PWS_binary)~Water_Area  +
                     TOPO_MEAN +
                     WS_Dens_mean +
                     StorBL +
                     DRT_WGT    +
                     Avg_Distance +
```

```
                     Cover_PctForest +
                     Cover_PctAgpast   +
                     PctProtected  +
                     Cons_spending   +
                     Cons_IUCN         +
                     CITY_POP           +
                     Gov_Index +
                     Enforcing.Contracts.Indicator  +
                     Natl_GDP_per_capita_Curr   +
                     AvgAnnGrowth     +
                     Registering.Property,
                   data=GwgtTrain,
                   weights = GwgtTrain$weights,
                   controls=cforest_unbiased(ntree=4000,mtry=4,trace=T))
cforestImpDotPlot(gwgtfit)
cforestImpBarPlot(gwgtfit)

#####  Model statistics and predictions  #####
gwgtfit.predict  <- predict(gwgtfit, GwgtTest, OOB=TRUE)
head(gwgtfit.predict)
  # run the following code to find the percent of predictions that were correct.
sum(GwgtTest$PWS_binary == gwgtfit.predict)/nrow(GwgtTest)
  # Confusion Matrix and % Misclassification Error
confusionMatrix(gwgtfit.predict, GwgtTest$PWS_binary)
  # Misclassification Error %
mean (GwgtTest$PWS_binary!= gwgtfit.predict)
  # c-static and somers dxy
Gwgtfit.trp <- treeresponse(gwgtfit, newdata=GwgtTest)
Gwgtfit.predforest <- sapply(Gwgtfit.trp, FUN = function(v) return(v[1]))
somers2(Gwgtfit.predforest, y=(as.numeric(GwgtTest$PWS_binary)))
```

Model Evaluation

```
### Model Evaluation
#Steps for ROC Curve and AUC calculation
# Use the model to predict the evaluation.
GwgtTest$prediction <- predict(gwgtfit, newdata=GwgtTest)
# Extract the class probabilities.
GwgtTest$probabilities <- 1- unlist(treeresponse
                                    (gwgtfit,
                                    newdata=GwgtTest),
                                    use.names=F)[seq(1,nrow(GwgtTest)*2,2)]
# Plot the performance of the model applied to the evaluation set as
# an ROC curve.
require(ROCR)
gwgtfit.pred <- prediction(GwgtTest$probabilities, GwgtTest$PWS_binary)
gwgtfit.perf <- performance(gwgtfit.pred,"tpr","fpr")
gwgtfit.auc.perf = performance(gwgtfit.pred, measure = "auc")
gwgtfit.auc.perf@y.values # this just prints out the AUC so I can look at it real quick
plot(gwgtfit.perf,
     main=paste('ROC CURVE, AUC:',round(gwgtfit.auc.perf@y.values[[1]],digits = 4)),
     print.thres=TRUE,
```

```
     colorize=T)
abline(h=1,col='blue',lwd=2)
abline(h=0,col='red',lwd=2)
```

Initial Random Forest Code with final variables after Reducing Dimensionality + global city weights AUC = 0.7054

2.3 Initial Random Forest Code with final variables after Reducing Dimensionality + regional city weights

```
set.seed(127)

RwgtTrain$weights <- as.integer(RwgtTrain$PWS_binary+1)

rwgtfit <- cforest(as.factor(PWS_binary)~Water_Area  +
                     TOPO_MEAN +
                     WS_Dens_mean +
                     StorBL +
                     DRT_WGT    +
                     Avg_Distance +
                     Cover_PctForest +
                     Cover_PctAgpast   +
                     PctProtected  +
                     Cons_spending   +
                     Cons_IUCN         +
                     CITY_POP           +
                     Gov_Index +
                     Enforcing.Contracts.Indicator  +
                     Natl_GDP_per_capita_Curr   +
                     AvgAnnGrowth     +
                     Registering.Property,
                   data=RwgtTrain,
                   weights = RwgtTrain$weights,
                   controls=cforest_unbiased(ntree=4000,mtry=4,trace=T))
cforestImpDotPlot(rwgtfit)
cforestImpBarPlot(rwgtfit)
```

Model Evaluation

```
### Model Evaluation
#Steps for ROC Curve and AUC calculation
# Use the model to predict the evaluation.
RwgtTest$prediction <- predict(rwgtfit, newdata=RwgtTest)
# Extract the class probabilities.
RwgtTest$probabilities <- 1- unlist(treeresponse
                                     (rwgtfit,
                                     newdata=RwgtTest),
                                     use.names=F)[seq(1,nrow(RwgtTest)*2,2)]
# Plot the performance of the model applied to the evaluation set as
# an ROC curve.
require(ROCR)
```

```r
rwgtfit.pred <- prediction(RwgtTest$probabilities, RwgtTest$PWS_binary)
rwgtfit.perf <- performance(rwgtfit.pred,"tpr","fpr")
rwgtfit.auc.perf = performance(rwgtfit.pred, measure = "auc")
rwgtfit.auc.perf@y.values # this just prints out the AUC so I can look at it real quick
plot(rwgtfit.perf,
     main=paste('ROC CURVE, AUC:',round(rwgtfit.auc.perf@y.values[[1]],digits = 4)),
     print.thres=TRUE,
     colorize=T)
abline(h=1,col='blue',lwd=2)
abline(h=0,col='red',lwd=2)
```

Initial Random Forest Code with final variables after Reducing Dimensionality + regional city weights AUC = 0.6549

2.4 Random Forest with SMOTE Balanced Data

```r
 # Lets look at the unbalance we're dealing with
print(table(PWS_gwgt$PWS_binary)) # Full Data
print(prop.table(table(PWS_gwgt$PWS_binary))) # Full Data
print(table(GwgtTrain$PWS_binary)) # Train Data
print(prop.table(table(GwgtTrain$PWS_binary))) # Train Data
print(table(GwgtTest$PWS_binary)) # Test Data
print(prop.table(table(GwgtTest$PWS_binary))) # Test Data

  # create balance using SMOTE to make some fake data
  # perc.over = 100 to double the quantity of positive cases
  # perc.under = 200 to keep half of what was created as negative cases.
GwgtTrain$PWS_factor <- as.factor(GwgtTrain$PWS_binary) #smote doesn't like to wrap the as.factor funct
gwgt_SMOTE <- SMOTE(PWS_factor ~ .,
                    data = GwgtTrain,
                    perc.over = 100,
                    perc.under = 750)
table(gwgt_SMOTE$PWS_factor) # check that there are now the same number of cases

  #random forest with the SMOTE output
set.seed(83)

gwgt_SMOTE$weights <- as.integer(gwgt_SMOTE$PWS_binary+1)

gwgt_SMOTEfit <- cforest(PWS_factor~Water_Area  +
                     TOPO_MEAN +
                     WS_Dens_mean +
                     StorBL +
                     DRT_WGT    +
                     Avg_Distance +
                     Cover_PctForest +
                     Cover_PctAgpast   +
                     PctProtected  +
                     Cons_spending   +
                     Cons_IUCN         +
                     CITY_POP            +
                     Gov_Index +
```

```
                        Enforcing.Contracts.Indicator  +
                        Natl_GDP_per_capita_Curr   +
                        AvgAnnGrowth     +
                        Registering.Property,
                      data=gwgt_SMOTE,
                      weights = gwgt_SMOTE$weights,
                      controls=cforest_unbiased(ntree=8000,mtry=4,trace=T))
cforestImpDotPlot(gwgt_SMOTEfit)
cforestImpBarPlot(gwgt_SMOTEfit)
```

Model Evaluation

```
#Steps for ROC Curve and AUC calculation
# Use the model to predict the evaluation.
SMOTETest <-GwgtTest
SMOTETest$prediction <- predict(gwgt_SMOTEfit, newdata=SMOTETest)
# Extract the class probabilities.
SmoteTest$probabilities <- 1- unlist(treeresponse
                                    (gwgt_SMOTEfit,
                                    newdata=SMOTETest),
                                    use.names=F)[seq(1,nrow(SMOTETest)*2,2)]
# Plot the performance of the model applied to the evaluation set as
# an ROC curve.
require(ROCR)
gwgt_SMOTEfit.pred <- prediction(SMOTETest$probabilities, SMOTETest$PWS_binary)
gwgt_SMOTEfit.perf <- performance(gwgt_SMOTEfit.pred,"tpr","fpr")
SMOTE.auc.perf = performance(gwgt_SMOTEfit.pred, measure = "auc")
SMOTE.auc.perf@y.values # this just prints out the AUC so I can look at it real quick
plot(gwgt_SMOTEfit.perf,
     main=paste('ROC CURVE, AUC:',round(SMOTE.auc.perf@y.values[[1]],digits = 4)),
     print.thres=TRUE,
     colorize=T)
abline(h=1,col='blue',lwd=2)
abline(h=0,col='red',lwd=2)
```

Random Forest with SMOTE Balanced Data AUC: 0.7054

2.5 Random Forest with Downsampled Data

Create Training and Test Data

```
set.seed(3456)

GwgtTrain1 <- GwgtTrain

GwgtTrain1$weights <- as.integer(GwgtTrain1$PWS_binary+1)

GwgttrainIndex <- createDataPartition(PWS_gwgt$PWS_binary, p = .8,
                                      list = FALSE,
                                      times = 1)
GwgtTrain1 <- PWS_gwgt[ GwgttrainIndex,]
```

```
GwgtTest1  <- PWS_gwgt[-GwgttrainIndex,]

table(GwgtTrain1$PWS_binary)
table(GwgtTest1$PWS_binary)
```

Random Forest with downsampled data

```
# Create downsample

D_Train1 <- downSample(x = GwgtTrain1, y = as.factor(GwgtTrain1$PWS_binary))
table(D_Train1$PWS_binary)

gwgt_dfit1 <- cforest(as.factor(PWS_binary)~Water_Area  +
                            TOPO_MEAN +
                            WS_Dens_mean +
                            StorBL +
                            DRT_WGT    +
                            Avg_Distance +
                            Cover_PctForest +
                            Cover_PctAgpast   +
                            PctProtected   +
                            Cons_spending   +
                            Cons_IUCN          +
                            CITY_POP            +
                            Gov_Index +
                            Enforcing.Contracts.Indicator  +
                            Natl_GDP_per_capita_Curr   +
                            AvgAnnGrowth     +
                            Registering.Property,
                          data=D_Train1,
                          weights = D_Train1$weights,
                          controls=cforest_unbiased(ntree=8000,mtry=4,trace=T))
cforestImpDotPlot(gwgt_dfit1)
print(gwgt_dfit1)
```

Model Evaluation

```
#Steps for ROC Curve and AUC calculation
# Use the model to predict the evaluation.
GwgtTest1$prediction <- predict(gwgt_dfit1, newdata=GwgtTest1)
# Extract the class probabilities.
GwgtTest1$probabilities <- 1- unlist(treeresponse
                                      (gwgt_dfit1,
                                      newdata=GwgtTest1),
                                      use.names=F)[seq(1,nrow(GwgtTest1)*2,2)]
# Plot the performance of the model applied to the evaluation set as
# an ROC curve.
require(ROCR)
gwgt_dfit1.pred <- prediction(GwgtTest1$probabilities, GwgtTest1$PWS_binary)
gwgt_dfit1.perf <- performance(gwgt_dfit1.pred,"tpr","fpr")
fit1.auc.perf = performance(gwgt_dfit1.pred, measure = "auc")
```

```
fit1.auc.perf@y.values # this just prints out the AUC so I can look at it real quick
plot(gwgt_dfit1.perf,
     main=paste('ROC CURVE, AUC:',round(fit1.auc.perf@y.values[[1]],digits = 4)),
     print.thres=TRUE,
     colorize=T)
abline(h=1,col='blue',lwd=2)
abline(h=0,col='red',lwd=2)
```

Random Forest with Downsampled Data AUC = 0.604

2.6 Random Forest with Weighted Classes

```
# Create Weighted Class

GwgtTrain2 <- GwgtTrain
GwgtTrain2$weights <- as.integer(GwgtTrain2$PWS_binary+1)

gwgt_dfit2 <- cforest(as.factor(PWS_binary)~Water_Area  +
                            TOPO_MEAN +
                            WS_Dens_mean +
                            StorBL +
                            DRT_WGT    +
                            Avg_Distance +
                            Cover_PctForest +
                            Cover_PctAgpast   +
                            PctProtected  +
                            Cons_spending   +
                            Cons_IUCN         +
                            CITY_POP            +
                            Gov_Index +
                            Enforcing.Contracts.Indicator  +
                            Natl_GDP_per_capita_Curr   +
                            AvgAnnGrowth     +
                            Registering.Property,
                          data=GwgtTrain2,
                          weights=GwgtTrain2$weights,
                          controls=cforest_unbiased(ntree=8000,mtry=4,trace=T))
cforestImpDotPlot(gwgt_dfit2)
print(gwgt_dfit2)
```

Model Evaluation

```
#Steps for ROC Curve and AUC calculation
# Use the model to predict the evaluation.

GwgtTest2 <- GwgtTest

GwgtTest2$prediction <- predict(gwgt_dfit2, newdata=GwgtTest2)
# Extract the class probabilities.
GwgtTest2$probabilities <- 1- unlist(treeresponse
                                     (gwgt_dfit2,
```

```
                                        newdata=GwgtTest2),
                                        use.names=F)[seq(1,nrow(GwgtTest2)*2,2)]
# Plot the performance of the model applied to the evaluation set as
# an ROC curve.
require(ROCR)
gwgt_dfit2.pred <- prediction(GwgtTest2$probabilities, GwgtTest2$PWS_binary)
gwgt_dfit2.perf <- performance(gwgt_dfit2.pred,"tpr","fpr")
fit2.auc.perf = performance(gwgt_dfit2.pred, measure = "auc")
fit2.auc.perf@y.values # this just prints out the AUC so I can look at it real quick
plot(gwgt_dfit2.perf,
     main=paste('ROC CURVE, AUC:',round(fit2.auc.perf@y.values[[1]],digits = 4)),
     print.thres=TRUE,
     colorize=T)
abline(h=1,col='blue',lwd=2)
abline(h=0,col='red',lwd=2)
```

Random Forest with Weighted Classes AUC: 0.7007

Print the above plot but more fancy with new names and some colors

```
#rename the variables
install.packages("dplyr")
library("dplyr")
install.packages("plyr")
library("plyr")

#rename the variables
GwgtTest3 <- plyr::rename(GwgtTest2,
       replace = c("Water_Area"="Total.Watershed.Area",
                   "TOPO_MEAN"="Average.Elevation",
                   "WS_Dens_mean"="Average.Watershed.Population.Density",
                   "StorBL"="Total.Diversion.Volume",
                   "DRT_WGT"="Weighted.Drought.Vulnerability.Index",
                   "Avg_Distance"="Average.Distance",
                   "Cover_PctForest"="Percent.Forest.Cover",
                   "Cover_PctAgpast"="Percent.Agriculture.Cover",
                   "PctProtected"= "Percent.Protected.Watershed",
                   "Cons_spending"="Cons_spending",
                   "Cons_IUCN"="IUCN.Organizations.Per.Million.People",
                   "CITY_POP"="City.Population",
                   "Gov_Index"="Average.Governance.Indicators",
                   "Natl_GDP_per_capita_Curr"="National.GDP.per.capita",
                   "AvgAnnGrowth"="Average.Annual.Growth"))

GwgtTrain3 <- plyr::rename(GwgtTrain2,
       replace = c("Water_Area"="Total.Watershed.Area",
                   "TOPO_MEAN"="Average.Elevation",
                   "WS_Dens_mean"="Average.Watershed.Population.Density",
                   "StorBL"="Total.Diversion.Volume",
                   "DRT_WGT"="Weighted.Drought.Vulnerability.Index",
                   "Avg_Distance"="Average.Distance",
                   "Cover_PctForest"="Percent.Forest.Cover",
                   "Cover_PctAgpast"="Percent.Agriculture.Cover",
```

```
                   "PctProtected"= "Percent.Protected.Watershed",
                   "Cons_spending"="Cons_spending",
                   "Cons_IUCN"="IUCN.Organizations.Per.Million.People",
                   "CITY_POP"="City.Population",
                   "Gov_Index"="Average.Governance.Indicators",
                   "Natl_GDP_per_capita_Curr"="National.GDP.per.capita",
                   "AvgAnnGrowth"="Average.Annual.Growth"))


#Do the random forest
gwgt_dfit3 <- cforest(as.factor(PWS_binary)~Total.Watershed.Area  +
                            Average.Elevation +
                            Average.Watershed.Population.Density +
                            Total.Diversion.Volume +
                            Weighted.Drought.Vulnerability.Index    +
                            Average.Distance +
                            Percent.Forest.Cover +
                            Percent.Agriculture.Cover   +
                            Percent.Protected.Watershed  +
                            Average.Annual.Conservation.Spending   +
                            IUCN.Organizations.Per.Million.People         +
                            City.Population           +
                            Average.Governance.Indicators +
                            Enforcing.Contracts.Indicator  +
                            National.GDP.per.capita   +
                            Average.Annual.Growth     +
                            Registering.Property,
                          data=GwgtTrain3,
                          weights=GwgtTrain3$weights,
                          controls=cforest_unbiased(ntree=8000,mtry=4,trace=T))
cforestImpDotPlot(gwgt_dfit3)

# Create the Variable Importance measures vector
VIM <- varimpAUC(gwgt_dfit3,conditional=F)

#Color the variables according to "bins"

  # Create a vector so I can use it to make a legend:
Bins<- c("Biophysical","Economic","Governance","Sociocultural")

#rename the variables
install.packages("dplyr")
library("dplyr")

Rename.Vim <- rename

  # Create a dataframe to call for the colors
Colors <- as.data.frame(VIM) #my vector of variable importance measures
  Colors["Total.Watershed.Area",2] <- "darkgreen"
  Colors["Average.Elevation",2] <- "darkgreen"
  Colors["Average.Watershed.Population.Density",2] <- "mediumpurple4"
  Colors["Total.Diversion.Volume",2] <- "darkgreen"
  Colors["Weighted.Drought.Vulnerability.Index",2] <- "darkgreen"
```

```
  Colors["Average.Distance",2] <- "darkgreen"
  Colors["Percent.Forest.Cover",2] <- "darkgreen"
  Colors["Percent.Agriculture.Cover",2] <- "darkgreen"
  Colors["Percent.Protected.Watershed",2] <- "orange"
  Colors["Average.Annual.Conservation.Spending",2] <- "darkorange"
  Colors["IUCN.Organizations.Per.Million.People",2] <- "darkorange"
  Colors["City.Population",2] <- "mediumpurple4"
  Colors["Average.Governance.Indicators",2] <- "darkorange"
  Colors["Enforcing.Contracts.Indicator",2] <- "blue"
  Colors["National.GDP.per.capita",2] <- "blue"
  Colors["Average.Annual.Growth",2] <- "blue"
  Colors["Registering.Property",2] <- "darkorange"

avector <- as.vector(Colors$VIM)
dot.input <- as.vector(Colors[order(avector),])

par(mfrow=c(1,1))
dev.off()
dotchart(Colors$VIM[order(Colors$VIM)],
        cex=1.5,
          labels = row.names(dot.input),
          color=  dot.input$V2,
          main="Global Cities",
         xlab="Variable Importance \n(values to the right of the red dashed vertical line are important)
  abline(v = abs(min(v)),
         col = "red",
         lty = "longdash",
         lwd = 2)
  abline(v = 0, col = "gray")
  legend("bottomright",
         inset=.05,
         title="Enabling Condition Bins",
         cex=1,
         c(data=Bins),
         fill=c("darkgreen","blue","darkorange","mediumpurple4"),
         horiz=TRUE)
```

2.7 Partial Dependence Plots

These are used to visualize directionality of the relationships between variables or potential thresholds.

All cities

```
# Create a learner using the MLR package with our cforest model parameters
install.packages("mlr")
install.packages("party")
library(party)
library(mlr)
lrn <- makeLearner("classif.cforest", predict.type = "prob",
                   par.vals = list(ntree = 8000,
                                   mtry = 4,
                                   trace=T))
```

```r
RF.Task <- makeClassifTask(data = GwgtTrain3,
                           target = "PWS_binary",
                           weights=GwgtTrain3$weights)
fit <-  train(lrn, RF.Task)

# Create Partial Dependence Plots

Watershed.Area.Plot <- generatePartialDependenceData(fit, RF.Task, "Total.Watershed.Area")
plotPartialDependence(Watershed.Area.Plot, data = getTaskData(RF.Task))

Ag.Cover.Plot <- generatePartialDependenceData(fit, RF.Task, "Percent.Agriculture.Cover")
plotPartialDependence(Ag.Cover.Plot, data = getTaskData(RF.Task))


Avg.Elevation.Plot <- generatePartialDependenceData(fit, RF.Task, "Average.Elevation")
plotPartialDependence(Avg.Elevation.Plot, data = getTaskData(RF.Task))

Drt.Index.Plot <- generatePartialDependenceData(fit,
                                                RF.Task,
                                                "Weighted.Drought.Vulnerability.Index")
plotPartialDependence(Drt.Index.Plot, data = getTaskData(RF.Task))

Avg.Distance.Plot <- generatePartialDependenceData(fit,
                                                RF.Task,
                                                "Average.Distance")
plotPartialDependence(Avg.Distance.Plot, data = getTaskData(RF.Task))

Percent.Protected.Plot <- generatePartialDependenceData(fit,
                                                RF.Task,
                                                "Percent.Protected.Watershed")
plotPartialDependence(Percent.Protected.Plot, data = getTaskData(RF.Task))

Avg.Cons.Plot <- generatePartialDependenceData(fit,
                                                RF.Task,
                                                 "Average.Annual.Conservation.Spending")
plotPartialDependence(Avg.Cons.Plot, data = getTaskData(RF.Task))


City.Pop.Plot <- generatePartialDependenceData(fit,
                                                RF.Task,
                                                "City.Population")
plotPartialDependence(City.Pop.Plot, data = getTaskData(RF.Task))

Contracts.Plot <- generatePartialDependenceData(fit,
                                                RF.Task,
                                                "Enforcing.Contracts.Indicator")
plotPartialDependence(Contracts.Plot, data = getTaskData(RF.Task))


Avg.GDP.Plot <- generatePartialDependenceData(fit,
                                                RF.Task,
                                                "Average.Annual.Growth")
plotPartialDependence(Avg.GDP.Plot, data = getTaskData(RF.Task))
```

```r
Property.Plot <- generatePartialDependenceData(fit,
                                               RF.Task,
                                               "Registering.Property")
plotPartialDependence(Property.Plot, data = getTaskData(RF.Task))


#Plot All of the Partial Plots Together

pd.lst = generatePartialDependenceData(fit, RF.Task,
                                       c("Percent.Agriculture.Cover",
                                         "Percent.Protected.Watershed",
                                         "City.Population",
                                         "Weighted.Drought.Vulnerability.Index",
                                         "Average.Annual.Growth",
                                         "Average.Distance",
                                         "Average.Annual.Conservation.Spending",
                                         "Average.Elevation",
                                         "Enforcing.Contracts.Indicator",
                                         "Total.Watershed.Area",
                                         "Registering.Property"),
                                       FALSE)
plotPartialDependence(pd.lst)
```

Non USA Cities Only

```r
# Create a learner using the MLR package with our cforest model parameters
install.packages("mlr")
library(mlr)
lrn <- makeLearner("classif.cforest", predict.type = "prob",
                   par.vals = list(ntree = 8000,
                                   mtry = 4,
                                   trace=T))

RF.Task2 <- makeClassifTask(data = NonUSA_cities_Train2,
                            target = "PWS_binary",
                            weights=NonUSA_cities_Train2$weights)
fit2 <-  train(lrn, RF.Task2)

# Create Partial Dependence Plots

Watershed.Area.Plot2 <- generatePartialDependenceData(fit2, RF.Task2, "Total.Watershed.Area")
plotPartialDependence(Watershed.Area.Plot2, data = getTaskData(RF.Task2))

Ag.Cover.Plot2 <- generatePartialDependenceData(fit, RF.Task2, "Percent.Agriculture.Cover")
plotPartialDependence(Ag.Cover.Plot2, data = getTaskData(RF.Task2))


Avg.Elevation.Plot2 <- generatePartialDependenceData(fit2, RF.Task2, "Average.Elevation")
plotPartialDependence(Avg.Elevation.Plot2, data = getTaskData(RF.Task2))

Drt.Index.Plot2 <- generatePartialDependenceData(fit2,
                                                 RF.Task2,
                                                 "Weighted.Drought.Vulnerability.Index")
```

```r
plotPartialDependence(Drt.Index.Plot2, data = getTaskData(RF.Task2))

Avg.Distance.Plot2 <- generatePartialDependenceData(fit2,
                                                 RF.Task2,
                                                 "Average.Distance")
plotPartialDependence(Avg.Distance.Plot2, data = getTaskData(RF.Task2))

Percent.Protected.Plot2 <- generatePartialDependenceData(fit2,
                                                 RF.Task2,
                                                 "Percent.Protected.Watershed")
plotPartialDependence(Percent.Protected.Plot2, data = getTaskData(RF.Task2))

Avg.Cons.Plot2 <- generatePartialDependenceData(fit2,
                                                 RF.Task2,
                                                  "Average.Annual.Conservation.Spending")
plotPartialDependence(Avg.Cons.Plot2, data = getTaskData(RF.Task2))


City.Pop.Plot2 <- generatePartialDependenceData(fit2,
                                                 RF.Task2,
                                                 "City.Population")
plotPartialDependence(City.Pop.Plot2, data = getTaskData(RF.Task2))

Contracts.Plot2 <- generatePartialDependenceData(fit2,
                                                 RF.Task2,
                                                 "Enforcing.Contracts.Indicator")
plotPartialDependence(Contracts.Plot2, data = getTaskData(RF.Task2))


Avg.GDP.Plot2 <- generatePartialDependenceData(fit2,
                                                 RF.Task2,
                                                 "Average.Annual.Growth")
plotPartialDependence(Avg.GDP.Plot2, data = getTaskData(RF.Task2))

Property.Plot2 <- generatePartialDependenceData(fit2,
                                                 RF.Task2,
                                                 "Registering.Property")
plotPartialDependence(Property.Plot2, data = getTaskData(RF.Task2))


#Plot All of the Partial Plots Together

pd.lst2 = generatePartialDependenceData(fit2, RF.Task2,
                                       c("Percent.Agriculture.Cover",
                                         "Percent.Protected.Watershed",
                                         "IUCN.Organizations.Per.Million.People",
                                         "Average.Elevation",
                                         "Average.Annual.Growth",
                                         "Enforcing.Contracts.Indicator",
                                         "Percent.Forest.Cover",
                                         "City.Population",
                                         "Registering.Property",
                                         "National.GDP.per.capita",
```

```r
                                                  "Average.Annual.Conservation.Spending",
                                                  "Average.Governance.Indicators"
                                                   ),
                                                FALSE)
plotPartialDependence(pd.lst2)
```
